# The first annotated set of scanning electron microscopy images for nanoscience

**DOI:** 10.1038/sdata.2018.172

**Published:** 2018-08-28

**Authors:** Rossella Aversa, Mohammad Hadi Modarres, Stefano Cozzini, Regina Ciancio, Alberto Chiusole

**Affiliations:** 1 CNR-IOM Istituto Officina dei Materiali, c/o SISSA, via Bonomea 265, 34136 Trieste, Italy; 2 Institute for Manufacturing, Department of Engineering, University of Cambridge, 17 Charles Babbage Road, Cambridge CB3 0FS, UK; 3 eXact-Lab srl, via Beirut 2, 34151 Trieste, Italy; 4 CNR-IOM, TASC Laboratory, Area Science Park, S.S.14, Km 163.5, 34149 Trieste, Italy

**Keywords:** Computational nanotechnology, Scientific data, Scanning electron microscopy

## Abstract

In this paper, we present the first publicly available human-annotated dataset of images obtained by the Scanning Electron Microscopy (SEM). A total of roughly 26,000 SEM images at the nanoscale are classified into 10 categories to form 4 labeled training sets, suited for image recognition tasks. The selected categories span the range of 0D objects such as particles, 1D nanowires and fibres, 2D films and coated surfaces as well as patterned surfaces, and 3D structures such as microelectromechanical system (MEMS) devices and pillars. Additional categories such as tips and biological are also included to expand the spectrum of possible images. A preliminary degree of hierarchy is introduced, by creating a subtree structure for the categories and populating them with the available images, wherever possible.

## Background & Summary

Access to data is playing an increasingly central role in research. Indeed, many research fields rely almost entirely on the availability, curation, and sharing of global data sources provided through data repositories, which have nowadays become a vital part of research infrastructures.

In this context the NFFA–EUROPE project (www.nffa.eu), an advanced European distributed research infrastructure dedicated to nanoscience, has, as one of its key tasks, the objective to design effective means of data sharing at the nanoscale in Europe. The project involves twenty European research partners, and more than 150 different experimental/computational instruments and techniques; the volume of scientific data produced by the project activities is aimed to be hosted in the NFFA-EUROPE Information and Data Repository Platform (IDRP)^1^ being open and fully available to the scientific community well defined data policy. The IDRP is complemented by a set of data analysis services that has been steadily growing since the beginning of the project.

Among the available instruments of the NFFA-EUROPE catalogue, Scanning Electron Microscopes (SEMs) are among the mostly requested ones from users, and are available at ten NFFA-EUROPE sites. SEM is indeed a routinely used characterization technique able to provide information about the samples' topography and composition down to a few nanometers resolution by scanning a focused electron beam onto the sample surface.

The first data analysis achievement is a service offered by the NFFA-EUROPE IDRP consisting of an automatic tool for image recognition to store, classify and label SEM images: we employed a supervised machine learning algorithm using deep convolutional neural networks for recognition of SEM images^2^. In order to train the network, it was necessary to provide a labeled training set, i.e. a set of SEM images properly classified by humans into categories.

In this paper, we describe the datasets presented in ref. 2, together with the methods employed to classify and validate them: the primary dataset used for neural network training (SEM dataset, Data Citation 1), a detached smaller dataset in which a hierarchical structure has been introduced (Hierarchical dataset, Data Citation 2), and two datasets which contain ~7,000 newly classified images in addition to the SEM dataset, according to different validation criteria (Majority dataset, Data Citation 3, and 100% dataset, Data Citation 4), as described in the Technical Validation section.

The images were generated by the SEM located at the CNR-IOM (Istituto di Officina dei Materiali, www.iom.cnr.it) in Trieste, Italy. In total, close to 100 users have generated roughly 150,000 images over five years at the facility.

From this sample, an initial batch of ~20,000 images was manually classified by nanoscientists into 10 categories which spanned a broad range of nano-objects, from 0D particles, 1D nanowires, 2D films and patterned surfaces, and 3D pillars and microelectromechanical devices.

This classified dataset (Data Citation 1) is intended to be functional to a number of possible implementations. Primarily, it can set the basis for a more complete labeled dataset for supervised machine learning which can be further expanded with more SEM images, thus allowing the development of an even more accurate and precise SEM image recognition software. Some categories can be split into multiple subcategories, as further discussed in the text.

On the other hand, image processing techniques are being increasingly applied to microscope images^3,4^. This dataset allows such programs to be tested on a large set of images belonging to a particular category for specific purposes.

In general, we are confident that the accuracy, diversity, and hierarchical structure of our SEM datasets can offer additional opportunities to researchers, connecting the image recognition and the nanoscience communities.

The paper is organized as follows: The Methods section describes how we retrieved and classified the SEM images; the Data Records section contains an overview of each dataset associated with this work, including the repository where they are stored, the contained data files, and their formats; in the Technical Validation section, we present the adopted validation procedure and the obtained results. Finally, the Usage Notes section offers a summary of the datasets and of the aims they were designed at.

## Methods

Most of the methods described in detail in this section have been presented in our previous publication^2^.

### SEM imaging

All the images collected in the datasets were acquired by nearly 100 scientists and researchers over five years using a ZEISS SUPRA 40 high resolution Field Emission Gun (FEG) SEM, located in the TASC laboratories of the CNR-IOM in Trieste. The microscope can be operated with 0.1–30 kV acceleration voltage and a 4 pA-10 nA probe current with a nominal resolution of 1.3 nm at 15 kV. It is equipped with an Everhart-Thornley Secondary Electrons Detector, a Back-scattered Electrons Detector, and a High efficiency In-lens detector, the latter providing an increased signal-to-noise ratio in image acquisition. The microscope is also equipped with a 4-Channel High-Angle Annular Scanning Transmission ElectronMicroscopy (STEM) detector allowing detection of transmitted electrons and with an Oxford Aztec Energy Dispersive X-ray Spectroscopy (EDS) system and a X-act 10 mm Silicon Drift Detector (SDD) for compositional analysis.

### SEM image classification

**Figure 1:**
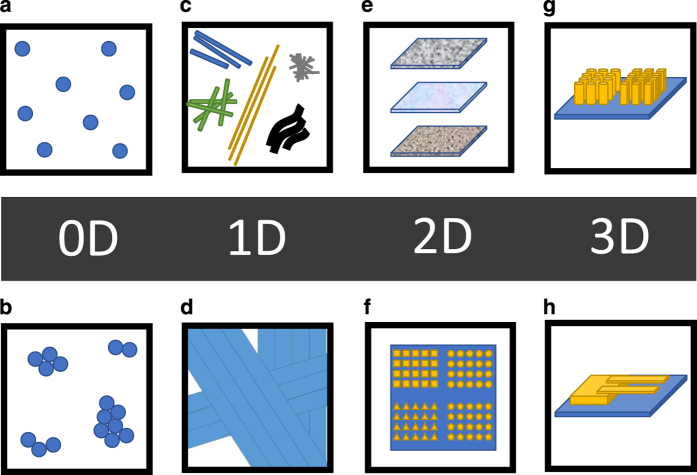
Schematic view of nanostructure classification based on dimensionality from 0D to 3D. (**a**) Particles (**b**) Clusters (**c**) Nanowires (**d**) Fibres (**e**) Films (**f**) Patterned surfaces (**g**) Pillars (**h**) MEMS devices.

The SEM images were classified based mainly on the dominant shape and structure of the imaged object. The dimensionality of the nanostructure (0D, 1D, 2D or 3D) was used as a first criterion, being the visual benchmark of the nanostructure itself. In this classification, 0D objects refer to particles which can be either dispersed and individually identified over the image area, or packed together into a clustered structure. 1D objects are referred to as nanowires, rods, or fibres. These structures can be either entangled in bundles or mutually aligned with respect to each other. 2D structures refer to films and coatings on a surface, and include a wide range of cases differing from each other for the surface roughness. 3D structures can refer to pillars or other micro-devices such as microelectromechanical systems (MEMS), typically fabricated by lithography techniques. A schematic view of this classification is illustrated in Fig. 1.

**Figure 2:**
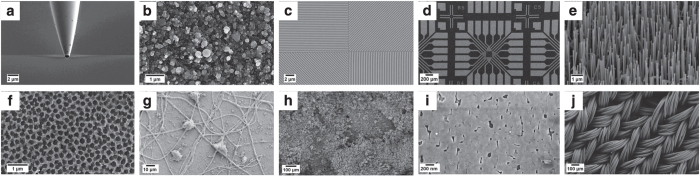
Representative images for each of the categories chosen for the SEM dataset. (**a**) Tips (**b**) Particles (**c**) Patterned surfaces (**d**) MEMS devices and electrodes (**e**) Nanowires (**f**) Porous sponge (**g**) Biological (**h**) Powder (**i**) Films and coated surfaces (**j**) Fibres. The dimensionality of nanoscience objects provided the basis for the choice. Other categories, such as Biological and Tips, were added, as they were common images found in the SEM database.

The dimensionality discussed above provided the main basis for categorization, but was not exhaustive. After investigation of the SEM images database and discussions with SEM scientists, additional categories such as biological samples and tips (these last mostly related to atomic force microscopes) were introduced to enlarge the spectrum of the possible categories. The full set of selected categories is illustrated in Fig. 2, with a representative image for each of them, while the breakdown of the number of images in each category is shown in Table 1. These ten categories cover a broad range of SEM images, and they are applicable to scientists working in different areas of nanoscience. At the same time, they were chosen to be as more distinct from one another as possible.

### Preliminary degree of hierarchy

A second dataset of 1,038 images (Data Citation 2) was selected to explore a subtree for each category, when appropriate. We warn that this must be considered a preliminary work, due to the very small number of images in ~40% of the subcategory nodes. A summary of this initial work is shown in Table 2. The table must be read from the left column to the right one as root-to-leaf branches. Images in some categories did not present a sufficient variety to be split into subclasses. In these cases, the columns of Level 1 and Level 2 were left empty. However, we do not exclude the possibility to enrich the dataset with new subcategories in the future, when more images will be analyzed.

### Code availability

The Python script used to generate the Majority dataset (Data Citation 3) and the 100% dataset (Data Citation 4) includes the following steps:

1. read the database containing the validation results from the web classifier (sem-classifier.nffa.eu) we developed;
2. extract the list of 400 images, distinguishing between the 200 images of the human set and the 200 images of the image recognition set;
3. count the validation results by the users for each image;
4. collect the images which have been validated by all the scientists and those which have been validated by the absolute majority of them (at least four out of seven);
5. create the 100% and the majority datasets, starting from the SEM dataset (Data Citation 1):
6. to create the Majority Dataset (Data Citation 3), images from the human set which were not validated by the absolute majority of the scientists were removed from the SEM dataset, while those from the image recognition set which were validated by the absolute majority of them were added;
7. to create the 100% dataset (Data Citation 4), images from the human set which were not validated by all of the scientists were removed from the SEM dataset, while those from the image recognition set which were validated by all of them were added.
8. The script can be provided by the authors upon request to Dr. Rossella Aversa (aversa@iom.cnr.it) or Dr. Stefano Cozzini (cozzini@iom.cnr.it).

## Data Records

In this section, we provide detailed information on the published datasets, including a breakdown of each of them. In addition to the original SEM dataset, we present a preliminary hierarchical subset and two increased datasets obtained by classifying and validating other ~7,000 images. A schematic description to summarize the main details is reported in Table 3, while validation results are described in the Technical Validation section and summarized in Table 4.

### SEM dataset

To guarantee the reproducibility of the convolutional neural network training results results presented in ref. 2, we provide the reference to the original SEM dataset (Data Citation 1) used in the paper. The SEM dataset consists of 18,577 SEM images produced at the CNR-IOM (Trieste, Italy). The images are 1,024×728 pixels jpg files, classified into 10 categories in a folder structure (Table 1). The folders are individually stored as tar archives, for a total size of ~10 GB, and are publicly available for download (Data Citation 1).

### Hierarchical dataset

The Hierarchical dataset is composed of 1,038 SEM images produced at CNR-IOM (Trieste, Italy). The images are 1,024×728 pixels jpg files, classified into 10 categories and 27 subcategories (as sub-trees) in a folder-subfolder structure (Table 2). The root folders are individually stored as tar archives, including the subfolders, for a total size of ~1 GB, and are publicly available for download (Data Citation 2). The preliminary results obtained from this dataset have been published in ref. 2.

### Majority dataset

The Majority dataset consists of 25,537 SEM images produced at CNR-IOM (Trieste, Italy). The images are 1,024×728 pixels jpg files, classified into 10 categories in a folder structure according to the majority criterion (Table 4): classification labels have been checked by a group of nanoscientists by means of our SEM web classifier (sem-classifier.nffa.eu), and only those images which have been validated by the absolute majority of the group have been included in the dataset. The folders are individually stored as tar archives, for a total size of 11 GB, and are publicly available for download (Data Citation 3).

### 100% dataset

The 100% dataset is composed of 25,430 SEM images produced at CNR-IOM (Trieste, Italy). The images are 1,024×728 pixels jpg files, classified into 10 categories in a folder structure according to the 100% criterion (Table 4): classification labels have been checked by a group of nanoscience experts by means of our SEM web classifier (sem-classifier.nffa.eu), and only those images which have been validated by all the members of the group have been included in the dataset. The folders are individually stored as tar archives, for a total size of ~10 GB, and are publicly available for download (Data Citation 4).

## Technical Validation

In this section, the procedure applied to validate the two increased SEM datasets and the statistical results obtained are presented. Since October 2017, we have labeled other ~7,000 images, thus increasing the dataset size by ~37%. As explained later, the method we applied ensures the same conclusions to be true also on the original SEM dataset (Data Citation 1).

To provide a reliable validation, 200 images (20 for each category) were randomly selected out to form a set (which we call human set) aimed to be judged by a group of 7 scientists, expert in SEM image interpretation. The human set was incremented by picking the same number of images (which we call image recognition set) classified by a neural network we had previously trained (see ref. 2 for details). We chose only image recognition results with a score ≥ 99%. We developed a web site (sem-classifier.nffa.eu), where the scientists were asked to access and validate the label assigned to each of the images or to suggest a different category, being unaware of which set (human or image recognition) they were coming from.

The dataset was validated at two levels: if a label of the human set reached the absolute majority of agreement from scientists, the image was validated (Majority dataset, Data Citation 3). This is a commonly adopted method, used e.g. to validate ImageNet^5^; alternatively, a more stringent criterion was to validate only the images for which the label reached the agreement of all the scientists in the group (100% dataset, Data Citation 4). By definition, the majority dataset contains the 100% dataset.

The results of the validation analysis are reported in Table 4. Looking at the last two rows, it can be noted that the images validated by the absolute majority of the scientists are almost 80% of the total (311 over 400), 168 (54%) of which come from the human set and 143 (45%) from the image recognition set. On the other side, roughly 50% of images (204 over 400) were validated by all the scientists; also in this case, more than 50% of validated images come from the human set (115 over 204).

It is worth noting that during the validation procedure 48 out of the 200 human labeled images were tagged by nanoscientists as “Not clear”. They should not be considered as misclassified images, rather they can be used to estimate the human uncertainty contribution to the validation error.

Based on the above results, the appropriate error on each of the two validated datasets can be expressed as:

(1)1−validatedtot−(nc-vnc)tot

The second fraction represents the human uncertainty, where nc (not clear) are all the images tagged as not clear, and vnc (validated not clear) images are those ones which have been tagged as not clear but still validated, thus included in the dataset (i.e., tagged as not clear by the minority of the scientists). Of course, vnc=0 when considering the 100% criterion.

According to this formula, we have

(2)1−168200−(48−25)200=0.045

for the Majority dataset, and

(3)1−115200−48200=0.185

for the 100% dataset. These errors, being calculated on a randomly selected set of 200 images labeled by humans, can be safely considered as the validation error on the total datasets.

To conclude, the datasets we present in this paper have a 4.5% validation error according to the most commonly adopted majority criterion, and a 18.5% validation error when a more stringent 100% criterion is applied.

If a label was not validated (according to the first or second criterion), the corresponding image was removed. The additional validated images from the image recognition set were added to the respective datasets (Majority or 100%), to increase the number of examples and eventually enhance the performance of future neural network trainings. Nevertheless, they were not included in the original SEM dataset (Data Citation 1), to guarantee the reproducibility of the scientific results presented in ref. 2.

## Usage Notes

We made available the four datasets presented in this paper through the EUDAT-B2SHARE service (https://b2share.eudat.eu/):

1. The original SEM dataset(Data Citation 1), to reproduce the results of ref. 2;
2. The Hierarchical dataset (Data Citation 2), which should be used carefully, being a preliminary version with few examples;
3. The Majority dataset (Data Citation 3), based on the extended SEM dataset. Images from the human set which were not validated by the absolute majority of scientists were removed, while images from the image recognition set validated by the absolute majority of scientists were added, to further increase the dataset size;
4. The 100% dataset (Data Citation 4), based on the extended SEM dataset. Images from the human set which were not validated by all of the nanoscientists were removed, while those from the image recognition set which were validated by all of the nanoscientists were added.

When applying machine learning techniques on these datasets, as a general rule, we suggest to use 90% of the images as the training set and 10% as the test set^6^.

All the datasets contain real SEM images as jpeg files, not as original tif format generated by the instrument. This implies that any scientific metadata associated to the measure is not present. By means of this choice, the authors make clear that the SEM image datasets are appropriate for the purposes of this study and in general for visual object recognition software research. Each dataset is therefore relevant as a whole, being the single images entirely detached from any specific information or scientific detail related to the displayed subject.

## Additional information

**How to cite this article**: Aversa, R. *et al*. The first annotated set of scanning electron microscopy images for nanoscience. *Sci. Data* 5:180172 doi: 10.1038/sdata.2018.172 (2018).

**Publisher’s note**: Springer Nature remains neutral with regard to jurisdictional claims in published maps and institutional affiliations.

## Supplementary Material



## Figures and Tables

**Table 1:** The original SEM annotated dataset.

**Category**	**N images**
Porous Sponge	171
Patterned surface	3,310
Particles	3,412
Films and Coated Surface	308
Powder	895
Tips	1,561
Nanowires	3,656
Biological	953
MEMS devices and electrodes	4,158
Fibres	153
TOTAL	18,577
The breakdown of the number of images in each category, and the total number of images composing the dataset are reported.	

**Table 2:** Preliminary hierarchical structure established.

**Category**	**Level 1 subcategory**	**Level 2 subcategory**	**N of images**
Tips	Zoom out		40
Tips	Zoom in		40
Tips	Tip on cantilever		40
Nanowires	Entangled nanowires		40
Nanowires	Few		35
Nanowires	Individual		30
Nanowires	Forest	Parallel aligned	30
Nanowires	Forest	Crust	25
Fibres			40
Biological			53
MEMS devices electrodes	Electrode		30
MEMS devices electrodes	Close up line		30
MEMS devices electrodes	3d edges		18
MEMS devices electrodes	Waveguide		8
MEMS devices electrodes	Microfluidic		10
Patterned surface	Line array		40
Patterned surface	Square array		20
Patterned surface	Circle array		60
Patterned surface	3d edge patterned surface		28
Patterned surface	3d line		50
Patterned surface	Triangle array		8
Patterned surface	Ring spiral		5
Patterned surface	Zigzag		5
Patterned surface	Pillars	3d array	20
Patterned surface	Pillars	Zoom in pillar	10
Patterned surface	Pillars	Individual pillar	15
Patterned surface	Pillars	Triangular pillar	10
Particles	Dispersed		40
Particles	Small clusters		25
Particles	Individual particle		15
Particles	Other shape		20
Powder	Zoom out		40
Powder	Zoom in		30
Films and coated surfaces	Smooth film		60
Films and coated surfaces	Particle film		35
Films and coated surfaces	Other film		40
Porous Sponge			13
Columns 1, 2, and 3 indicate the root of the subtree, the first, and the second level of nodes, respectively. The last column reports the number of images in the last node.			

**Table 3:** Summary of the datasets presented in this paper.

**Dataset**	**Categories**	**Images**	**Description**
SEM	10	18,577	Dataset used in ref. 2
Hierarchical	37	1,038	Hierarchical, preliminary subset
Majority	10	25,537	Augmented, validated applying majority criterion
100%	10	25,430	Augmented, validated applying 100% criterion
For each dataset, named in column 1, the total number of categories (or subcategories), the total number of images, and a short description are reported in columns 2, 3, and 4, respectively.			

**Table 4:** Validation results, according to both majority and 100% criteria.

**Category**	**Majority criterion**		**100% criterion**	
	human [%]	machine [%]	human [%]	machine [%]
Porous Sponge	95	50	75	35
Patterned surface	55	80	35	65
Particles	85	60	30	15
Films and Coated Surface	85	75	40	35
Powder	65	80	35	15
Tips	100	55	95	45
Nanowires	75	95	60	85
Biological	100	80	75	65
MEMS devices and electrodes	95	80	75	65
Fibres	85	60	55	30
PARTIAL TOTAL	168	143	115	89
TOTAL PER CRITERION	311		204	
The numbers of validated images are expressed in percentage with respect to the category size (20 images for each of them). The total numbers of images are reported in the last two rows.				
