# Author Correction: The first annotated set of scanning electron microscopy images for nanoscience

**DOI:** 10.1038/s41597-019-0007-8

**Published:** 2019-02-05

**Authors:** Rossella Aversa, Mohammad Hadi Modarres, Stefano Cozzini, Regina Ciancio, Alberto Chiusole

**Affiliations:** 1 CNR-IOM Istituto Officina dei Materiali, c/o SISSA, via Bonomea 265, 34136 Trieste, Italy; 2 Institute for Manufacturing, Department of Engineering, University of Cambridge, 17 Charles Babbage Road, Cambridge, CB3 0FS UK; 3 eXact-Lab srl, via Beirut 2, 34151 Trieste, Italy; 4 CNR-IOM, TASC Laboratory, Area Science Park, S.S.14, Km 163.5, 34149 Trieste, Italy

Correction to: *Scientific Data* 10.1038/sdata.2018.172, published online 28 August 2018

Following further analysis of the Majority Dataset (Data Citation 3, originally 10.23728/b2share.e344a8afef08463a855ada08aadbf352) and 100% Dataset (Data Citation 4, originally 10.23728/b2share.f1aa0f5ad38c456eaf7b04d47a65af53) presented in the original version of this Data Descriptor it was revealed that a large number of duplicate images were included in both datasets. Both datasets have been corrected in updated versions, removing all replicates. The new version of the Majority Dataset (Data Citation 3) can be accessed via 10.23728/b2share.72758204db9044ab8b3e6b6c4d2eb576 and the 100% Dataset (Data Citation 4) via 10.23728/b2share.80df8606fcdb4b2bae1656f0dc6db8ba. The HTML and PDF versions of the Data Descriptor have been corrected accordingly.

In addition, the authors found that the category ‘3d array’ was erroneously added to the Hierarchical Dataset (Data Citation 2). This has now been removed.

In order to account for the modifications to the datasets described above, a number of textual modifications to the HTML and PDF versions of Data Descriptor have been made. These are listed by their location below:

**Abstract**

The number of SEM images classified was previously stated as ~26,000. This has been correct to ~22,000.

**Background & Summary**

Where it was previously stated that the Majority Dataset and 100% Dataset contain ~7,000 newly classified images, the text has been corrected to state ~3,000 newly classified images.

**Data Records**

The two increased datasets were stated as being obtained by classifying and validating ~7,000 images. This has been corrected to ~3,000 images.

**Hierarchical dataset**

The number of subcategories into which images were classified has been corrected from 27 to 26.

**Majority dataset**

The number of SEM images in the Majority Dataset has been corrected from 25,537 to 21,272.

**100% dataset**

The number of SEM images in the 100% Dataset has been corrected from 25,430 to 21,169.

**Technical Validation**

Previously it was stated that ~7,000 images were labelled since October 2017, and the size of the dataset correspondingly increased by ~37%. This has been corrected to ~3,000 images and ~17%.

**Table 2**

Previously Table 2 included a row concerning the ‘3d array’ subcategory that was added erroneously. This has now been removed.

**Table 3**

The number of categories in the Hierarchical Dataset was corrected from 37 to 36. The number of images in the Majority and 100% datasets was corrected from 25,537 to 21,272 and from 25,430 to 21,169, respectively.

